# Pancreatic Pseudocyst with Splenic Artery Erosion, Retroperitoneal and Splenic Hematoma

**DOI:** 10.1155/2015/981860

**Published:** 2015-12-13

**Authors:** Petre V. H. Botianu, Adrian S. Dobre, Ana-Maria V. Botianu, Danusia Onisor

**Affiliations:** ^1^Surgical Clinic 4, M5 Department, University of Medicine and Pharmacy of Tirgu-Mures, 540141 Tirgu-Mures, Romania; ^2^Gastroenterology Clinic/Medical I, M3 Department, University of Medicine and Pharmacy of Tirgu-Mures, 540136 Tirgu-Mures, Romania; ^3^Gastroenterology Clinic/Medical VII, M3 Department, University of Medicine and Pharmacy of Tirgu-Mures, 540141 Tirgu-Mures, Romania

## Abstract

The erosion of the peripancreatic vascular structures is a rare but life-endangering complication of pancreatic diseases. We report a female patient with a multicompartmentalized pancreatic pseudocyst that eroded the splenic artery resulting in a retroperitoneal and splenic hematoma with hemodynamic instability which required emergency laparotomy with splenectomy, partial cystectomy, ligation of the splenic artery at the level of the vascular erosion, cholecystectomy (lithiasis), and multiple drainage. The postoperative course was difficult (elevated level of platelets, pancreatic fistula) but eventually favourable, with no abdominal complaints and no recurrence at 2-year follow-up. The case shows that the pancreatic pseudocysts may present with acute hemorrhagic complications with life-endangering potential and significant postoperative morbidity.

## 1. Introduction

Vascular complications are a common finding in benign pancreatic diseases. Most frequently the neighbourhood venous system is involved by inflammation and pressure resulting in thrombosis of the portal vein and its branches [1]. The erosion of the vascular structures with secondary bleeding is more rare but has a much higher life-endangering potential [2].

## 2. Case Report

**Figure 1:**
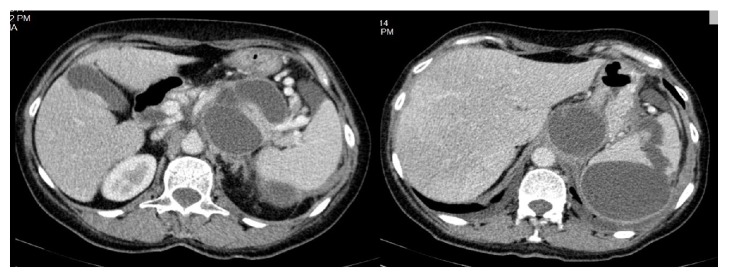
Preoperative CT scan showing 3 distinct cystic masses in the area of pancreas and spleen.

**Figure 2:**
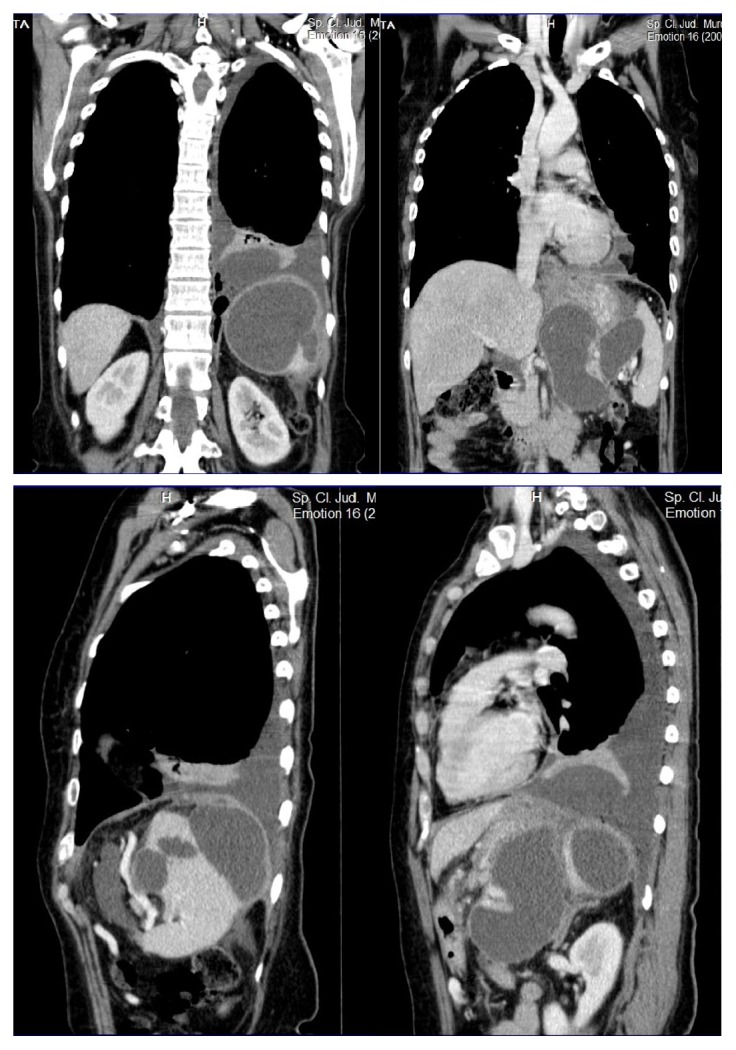
Preoperative CT scan: 3D reconstructions.

We report the case of a 58-year-old female patient with a history of acute pancreatitis with conservative treatment 2 years ago, chronic alcohol consumption with affirmative abandoning in the last 12 months, and an admission for a left pleural effusion one month ago, with resolution under antibiotic treatment. Due to the persistent abdominal pain, the patient underwent an abdominal ultrasound and CT scan which showed biliary lithiasis and the presence of 3 distinct large cystic masses in the pancreas and spleen (Figures 1 and 2). Surgery was proposed but the patient refused it.

The patient was subsequently admitted for intense abdominal pain with altered general status (blood pressure 90/60, pulse 110) and a drop of the hemoglobin level to 8.2 g/dL. An emergency CT scan showed the same cystic lesions and raised the suspicion of a ruptured splenic artery pseudoaneurysm and splenic hematoma. Due to the obvious signs of important bleeding and the impossibility to perform emergency arteriography, we decided to perform an emergency laparotomy.

**Figure 3:**
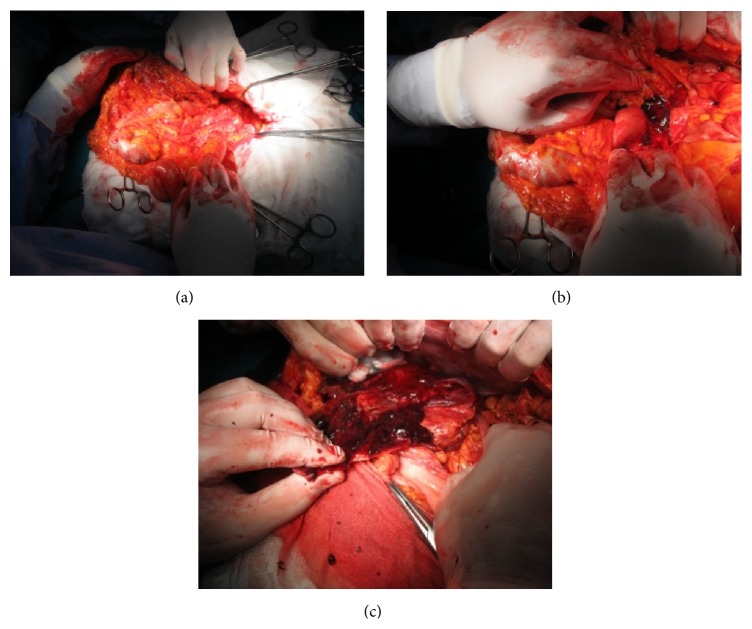
Intraoperative aspects: intraperitoneal blood, retroperitoneal and splenic hematoma.

**Figure 4:**
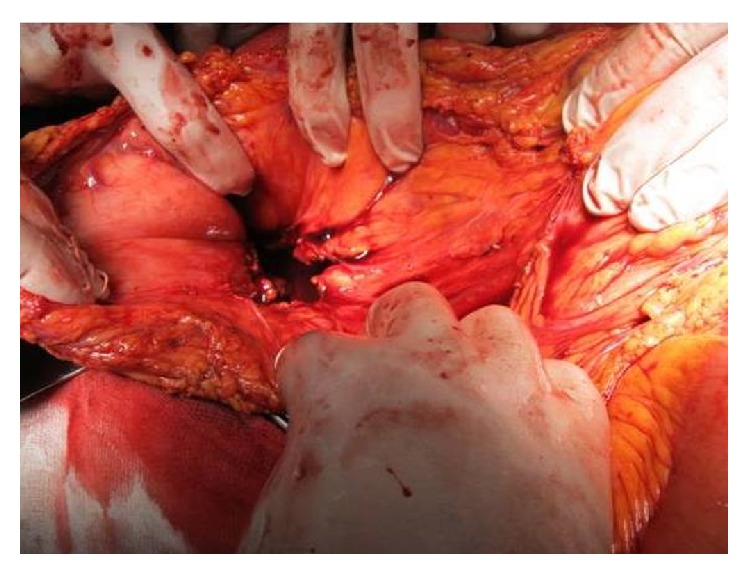
Intraoperative aspect: active pulsatile bleeding inside the cystic cavity.

**Figure 5:**
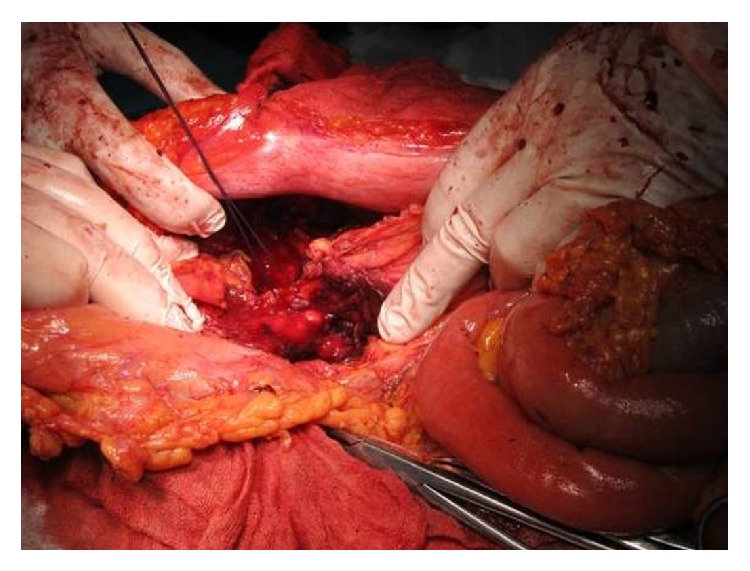
Intraoperative aspect: ligation of the splenic artery at the level of the vascular erosion.

Intraoperatively, we found a small amount of intraperitoneal blood and a large pancreatic pseudocyst filled with clots and fresh blood, extending into the retroperitoneum and spleen, corresponding to the 3 cystic lesions identified on the preoperative CT scan (Figure 3). An active pulsatile bleeding from the splenic artery was identified in the cyst cavity (Figure 4). Anatomic dissection of the splenic vessels at the level of the upper pancreatic border failed due to fibrosis and adhesions. We performed splenectomy, partial cystectomy with ligation of the splenic artery at the level of the vascular erosion (Figure 5), cholecystectomy (lithiasis), and multiple drainage.

The immediate postoperative course was favourable, with discharge on postoperative day 19. The patient presented 2 postoperative complications: an elevated level of platelets which required prolonged anticoagulation (low molecular weight heparin followed by oral warfarin treatment) and a pancreatic fistula with a 100–200 mL/day output which closed spontaneously after 5 months. At 2-year follow-up the patient presents with no abdominal complaints and no recurrence.

## 3. Discussions

The erosion of the vascular structures is a rare but serious complication of the pancreatic pseudocysts. All the neighbourhood vessels may be involved and the clinical course of this complication is determined by the anatomic location of the erosion and the rapidity of the blood losses. In most cases the bleeding will occur in the cavity of the pseudocyst, followed by retro- and/or intraperitoneal spillage of the blood [3, 4]. If a connection with the digestive lumen is established, the patient will present an upper digestive bleeding with hematemesis and/or melena [5, 6]. Other rare possibilities are the exteriorization of the blood through the pancreatic duct and the ampulla of Vater resulting in “hemosuccus pancreaticus” [7, 8] and the development of a splenic hematoma [9].

In the available literature, we found only case reports and small series of pancreatic pseudocysts complicated by erosion of vascular structures, which suggests a low incidence. A high proportion of the patients will require emergency laparotomy and have a significant postoperative morbidity, with fatal outcome being also possible [6, 10].

The optimal management of chronic pancreatitis complicated with vascular erosion is a matter of debate [11]. If the patient presents a stable hemodynamic status, endovascular approaches, placement of stents and/or embolisation, may be an elegant alternative to achieve hemostasis. In selected cases, endovascular and endoscopic approaches may be combined to solve both the vascular erosion and the cyst cavity [8, 12]. Open surgery is a solution if the patients present with hemodynamic instability and/or if the endovascular and endoscopic therapies are not available or have failed [11]. In our case, it is obvious that an earlier therapeutic intervention would have allowed a more conservative approach. A cystodigestive anastomosis would have avoided the two postoperative complications (increased platelet levels and the pancreatic fistula).

## 4. Conclusions

The erosion of the splenic artery is a possible evolutive complication of pancreatic pseudocysts and has a real life-endangering potential; it should be a serious argument for the active treatment (endoscopic, laparoscopic, or open) in large pancreatic pseudocysts. Emergency surgery may be required with hemostasis and treatment of the cyst cavity, carrying a significant morbidity.
